# Discrepancies of bovine haptoglobin concentrations between serum and plasma using two different anticoagulants and a colorimetric assay based on peroxidase activity

**DOI:** 10.1111/vcp.13386

**Published:** 2024-10-21

**Authors:** R. Schmitt, R. Staufenbiel

**Affiliations:** ^1^ Ruminant and Swine Clinic Free University of Berlin Berlin Germany

**Keywords:** acute‐phase protein, colorimetric assay, dairy cow, ELISA, haptoglobin, inflammation

## Abstract

**Background:**

Haptoglobin (Hp) is an emerging diagnostic marker in cattle, and knowledge of suitable sample types and measurement methods is important.

**Objectives:**

The aims of this study were to compare the results of a colorimetric assay (CA) and an ELISA for bovine Hp using serum, EDTA plasma, and lithium‐heparinized (LH) plasma, respectively, and to assess the diagnostic potential for puerperal metritis.

**Methods:**

In experiment 1, Hp was measured in pooled aliquots of serum (*n* = 10), EDTA plasma (*n* = 10), and LH plasma (*n* = 10) of 100 healthy fresh lactating dairy cows from 10 farms using both the CA and the ELISA. In experiment 2, five healthy and five cows with acute puerperal metritis were sampled, and Hp was determined using both assays for all three sample types. In experiment 3, aliquots of serum and LH plasma from cows in different lactation stages were transferred into plain, EDTA‐coated, and LH‐coated tubes and mixed before colorimetric analyses. Distilled water was also placed into each tube type and treated similarly.

**Results:**

Plasma samples measured with the CA showed on average 2.3 (EDTA) and 2.5 (LH) times higher Hp concentrations compared with serum, whereas no differences were seen with the ELISA results between sample types. Based on a clinical cut‐off value, both methods differentiated sick from healthy cows. Haptoglobin measurements with the ELISA were less precise compared with CA measurements due to high dilutions. No influence of the anticoagulants on the CA was observed.

**Conclusions:**

Due to measurement discrepancies between serum and plasma, CAs for bovine Hp based on peroxidase activity should be performed with serum, or specific reference ranges for plasma samples should be established. In this study, CA results obtained with LH plasma were more precise than results obtained with EDTA plasma. Both the CA and the ELISA are suitable diagnostic tools for the diagnosis of puerperal metritis, but CA measurements were more precise in this study.

## 1 INTRODUCTION

When facing different noxae, such as stress, pain, tissue injury, and infectious agents, the innate immune system reacts with an acute‐phase response. Macrophages and dendritic cells release proinflammatory cytokines (eg, IL‐1‐β, IL‐6, and TNF‐α), which, besides local effects, enhance the liver‐centered synthesis of acute‐phase proteins (APPs).^1^ Positive APPs accumulate in the blood and function in eliminating the respective noxae (ie, fighting invading pathogens and repairing injured tissues).^2^ Despite the high level of genetic homology among mammals, APPs are species‐specific in their diagnostic value.^3^ In bovine pathology today, one of the most important APPs frequently used in clinical diagnostics is the hemoglobin scavenger protein, haptoglobin (Hp).^2^, ^4^ Haptoglobin has been shown to have diagnostic value in detecting subclinical (and clinical) mastitis using milk samples^5^ and has recently proven useful in distinguishing between different mastitis‐causing pathogens.^6^ Measured in blood samples, Hp was further found to be associated with metritis and endometritis,^7^, ^8^ claw lesions,^9^ enzootic pneumonia,^10^ and other diseases of cattle. More recently, Hp was proposed to be both a prepartum^11^, ^12^ and early postpartum^13^, ^14^ predictive diagnostic marker for transition diseases in dairy cows. This is because Hp concentrations in blood mirror the degree of systemic inflammation postpartum, which precedes and likely promotes (or possibly causes) clinical disease.^15^, ^16^

Disease events associated with excessive inflammatory processes during the periparturient period of dairy cows negatively impact milk yield, milk composition, and reproductive performance following lactation, further harming the economic productivity of dairy farms.^17^ Therefore, the early detection of cows at risk for excessive postpartum inflammation could facilitate appropriate monitoring and treatment of affected animals, thereby potentially minimizing economic losses for dairy farms over time.

However, when it comes to using laboratory analytes in veterinary clinical diagnostics, practitioners and researchers need to rely on recommendations for suitable measurement techniques and sample types. Commonly used assays for bovine Hp are colorimetric assays (CAs)^18^, ^19^ and ELISAs.^20^, ^21^ Modern biosensors^22^, ^23^ and immunoturbidimetric assays^24^ are newly available and promising but still need to become established in clinical practice. It has been previously reported that different assays can have variable results,^25^ which might confuse practitioners trying to assess APPs as diagnostic markers. Furthermore, reference values for serum and plasma APP concentrations have been found to differ,^26^ which is critical when interpreting measurement results.

This study was conducted to compare Hp levels determined by two different commonly used measurement techniques (CAs and ELISAs) in serum and plasma from both lithium‐heparinized (LH) and EDTA tubes. Our hypotheses were that (1) Hp concentrations in serum, heparinized plasma, and EDTA plasma samples are the same and (2) Hp measurement results and the diagnostic capability of two commercially available assays, CAs and ELISAs, are equal.

## 2 MATERIALS AND METHODS

The experimental procedures were conducted with the approval of the Federal State Office of Occupational Safety, Consumer Protection and Health (animal care protocol number: 2347‐A‐3‐1‐2018).

To assess our hypotheses, three consecutive experiments were conducted. Experiment 1 was designed to compare the two methods and the three sample types. Experiment 2 was performed to validate observations from experiment 1 and assess the ability of the two methods and three sample types to diagnose puerperal metritis. Based on the results of experiments 1 and 2, experiment 3 was added to verify a possible anticoagulant interference with the CA.

### 2.1 Animals and farms

Blood samples used in experiment 1 were obtained from a total of 100 cows (*n* = 22 primiparous, *n* = 78 pluriparous) from 10 different commercial dairy farms that were enrolled in a larger clinical trial from March to April 2018. Detailed characteristics of these animals and farms are provided in the respective research article.^14^ Briefly, all farms were located in the northeast of Germany, kept ≥1000 Holstein Friesian dairy cows in free stall barns with cubicles, and fed their cows a total mixed ration. For experiments 2 (*n* = 10 cows) and 3 (*n* = 4 cows), samples were derived from a single farm that had also participated in experiment 1.

### 2.2 Clinical examinations and study enrollment

All cows used in this study were clinically examined for general health by assessing the general appearance and demeanor, body condition,^27^ rumen fill,^28^ locomotion score,^29^ and rectal body temperature (VT 1831 Veterinary Thermometer, Klifovet AG, Germany). In early postpartum cows, vulvovaginal laceration^30^ and quality of vaginal discharge (Metricheck Score^31^) were additionally scored. In experiment 1, only clinically healthy cows that had experienced a light calving process (no stillbirth, twin birth or dystocia, no assistance at calving) were enrolled within 8 days postpartum. In experiment 2, both healthy (*n* = 5) and sick (*n* = 5) cows were included within 8 days postpartum. Sick cows were suffering from acute puerperal metritis presenting with reduced appetite, signs of pain (eg, arched back, firm belly), reddish‐brownish, watery, foul‐smelling vaginal discharge, and elevated rectal body temperature ≥39.5°C.^32^ Four clinically healthy cows from different lactation stages (early postpartum [*n* = 1], midlactation [*n* = 1], late lactation [*n* = 1], and dry period [*n* = 1]) were used for experiment 3.

### 2.3 Sampling

Throughout the study, an experienced veterinarian collected all blood samples from the coccygeal vessel using an open blood collection system.

In experiment 1, three samples were obtained from each cow. The first was collected into a plain tube for separation of serum. The second and third were collected into vacutainer tubes containing EDTA and LH, respectively, for separation of plasma (tubes by SARSTEDT AG and Co. KG, Germany). Blood samples were allowed to clot for approximately 2 h. Plain and anticoagulant‐coated tubes were centrifuged for 10 min at 3500*g* and 2800*g*, respectively. For each farm and sample type, pooled samples were prepared. This process involved pipetting equal volumes (1 mL) from the 10 individual samples into a common pooling tube (10 mL). These pooled serums, LH, and EDTA plasma samples were subsequently divided into aliquots and stored at −24°C until analysis. Additionally, one individual serum sample from each farm was randomly chosen for blinded double determination in the laboratory to assess the analytical precision of the respective measurement method.

For experiment 2, from healthy cows and cows with acute puerperal metritis, respectively, three individual blood samples (serum, EDTA plasma, LH plasma) were obtained and processed as explained earlier, except that samples were not pooled.

In experiment 3, only two samples (serum, LH plasma) were collected from each cow. After centrifugation, individual aliquots (1 mL) from both sample types were transferred into three tubes (plain tube, EDTA‐coated tube, LH‐coated tube) and gently mixed. As a control, 1 mL aliquots of distilled water were aliquoted into three fresh sampling tubes (plain, EDTA‐coated, LH‐coated) and mixed.

### 2.4 Haptoglobin assays

Samples were sent to two accredited veterinary medical laboratories in Germany (DAkkS accreditation according to DIN EN ISO/IEC 17025:2018) providing two different measurement techniques. In experiments 1 and 2, concentrations of Hp were determined in aliquots of serum, EDTA plasma, and LH plasma, respectively, with both measurement techniques: a CA performed on a chemical autoanalyzer (Cobas 8.000 C 701 Autoanalyzer, Roche Diagnostics, Switzerland) using the “PHASE” Haptoglobin Assay Cat. No. TP‐801 (Tridelta Development, Ireland) and an ELISA test kit (BIO‐X‐Diagnostics S.A., Rochefort, Belgium). The colorimetric measurement, first described by Owen et al.,^18^ is based on the peroxidase activity of hemoglobin, which is usually inhibited by a low test pH, but can be preserved by Hp, if present in the specimen. The extinction read at 600–630 nm is directly proportional to the amount of Hp in the sample. The CA used in this study was calibrated according to the manufacturer's instructions using standardized PBS (0.00 g/L Hp) and 50 μL of a standard containing 2.5 g/L Hp from processed equine serum, which by 50 μL stepwise dilutions gives further calibrator solutions with 1.25, 0.625, and 0.312 g/L Hp, respectively. As controls, two different solutions with known Hp concentrations from processed equine serum provided by the manufacturer were included in every assay. The detection limit of the assay is officially declared as 0.005 g/L Hp based on two standard deviations added to the mean optical density determined from 32 measurements of the 0.00 g/L PBS calibrator solution. Serum and heparinized plasma samples are referred to as suitable sample types by the manufacturer of the CA. According to the present laboratory's quality control documentation of the CA, dilutions have never been needed to obtain reliable results in bovine Hp analyses.

The ELISA test kit uses monoclonal antibodies directed specifically against bovine Hp. As standard, lyophilized bovine haptoglobin is first dissolved in 500 μL distilled water and subsequently diluted with 1350 μL of an included dilution solution to give a 0.36 g/L Hp standard. Further stepwise (150 μL steps) dilutions result in further calibrator solutions containing 0.18, 0.09, 0.045, 0.0225, 0.01125, and 0.0056 g/L, respectively, representing the detection range of the ELISA as declared by the manufacturer (0.0056–0.36 g/L). A specific antibovine Hp antibody coupled to peroxidase is used as conjugate. The subsequent reaction of the chromogen tetramethylbenzidine (TMB) and peroxidase is stopped by acidification with phosphoric acid, and the optical densities are read at 450 nm. Initially, all bovine serum samples were diluted by factor 1000 to enter the detection range of the assay as instructed by the manufacturer. Samples that contained more than 0.36 g/L Hp were further diluted using the provided dilution buffer, starting with a 10‐fold dilution, and the ELISA measurement was repeated thereafter.

The mean intra‐ and interassay coefficients of variation officially declared by the laboratories were 1.2% and 5.3%, respectively, for the CA and 2.9% and 16.2%, respectively, for the ELISA. In experiment 1, blinded double determinations were performed from one randomly selected individual serum sample of each farm in order to verify the assays' analytical precision with the study data. In experiment 3, only the CA was used to measure Hp concentrations in distilled water, serum, and LH plasma samples, each previously mixed in three different types of tubes.

### 2.5 Statistical data analyses

Data for the experiments were collected in Microsoft Excel (Office 2013; Microsoft Deutschland Ltd., Munich, Germany). Statistical analyses were performed using SPSS for Windows (Version 26.0; SPSS Inc., Chicago, IL). Comparative analyses of sample types and measurement methods were conducted in alignment with recommendations by Jensen and Kjelgaard‐Hansen.^33^

### 2.6 Comparison of serum, EDTA plasma, and LH plasma

The relationships among Hp concentrations in serum, EDTA plasma, and LH plasma measured with the CA and the ELISA, respectively, were assessed using Pearson's correlation coefficient and univariable linear regression analysis suppressing an intercept. Measurement results were further compared between sample types with Friedman's test and Bland–Altman statistics.^34^

### 2.7 Comparison of the CA and ELISA techniques

Pearson's correlation coefficient was used to evaluate the general relationship between the measurement results of the CA and the ELISA. Using Wilcoxon's test, Hp concentrations were compared between the two methods. To further explain differences in Hp concentrations measured by the CA and the ELISA, Bland–Altman plots^34^ were created. Precision of both measurement techniques was assessed by calculating intra‐assay CV from duplicate serum analyses using the root mean square (RMS) method.^35^

### 2.8 Evaluation of the capability to distinguish between sick and healthy cows

Disease status was dichotomized as follows: 0 = healthy, 1 = sick (metritic). The Mann–Whitney *U* test was used to test for differences in Hp concentrations in all three sample types analyzed with the CA or ELISA test kit. Besides this statistical approach, a commonly used cut‐off value of 0.60 g/L for nonmetritic vs metritic cows in the early postpartum period^36^, ^37^ was also used to evaluate the capability of the methods and sample types to differentiate sick from healthy cows from a clinical diagnostic point of view.

### 2.9 Evaluation of possible influences of the anticoagulants on the colorimetric measurement

Haptoglobin concentrations determined with the CA in serum, LH plasma, and distilled water previously mixed in plain, EDTA‐coated, and LH‐coated tubes, respectively, were compared using Friedman's test.

### 2.10 Level of significance

Associations and differences were considered significant if *p* < .05 and tendencies were declared at .05 ≤ *p* ≤ .10.

## 3 RESULTS

Supporting information are provided in a Supplementary File.

### 3.1 Experiment 1

#### 3.1.1 Comparison of serum, EDTA plasma, and LH plasma

Significant differences in Hp concentrations between the three sample types were observed with the CA but not with the ELISA (Tables 1, 2, 3). Compared with serum, the CA revealed on average 2.3 and 2.5 times higher Hp concentrations in EDTA and LH plasma samples, respectively (Tables 1 and 3; *p* < .05).

**TABLE 1:** Haptoglobin (Hp) concentrations determined in pooled serum (*n* = 10), EDTA plasma (*n* = 10), and lithium‐heparinized (LH) plasma (*n* = 10) samples of 100 dairy cows within 0–8 days postpartum from 10 farms using a colorimetric assay (CA) on a chemical autoanalyzer and an ELISA, respectively.

	*n*		Hp (CA) (g/L)	Hp (ELISA) (g/L)
Serum	10	x¯	0.74^aA^	0.47^aB^
		SD	0.26	0.25
		Min	0.36	0.17
		Max	1.10	0.99
EDTA plasma	10	x¯	1.72^bA^	0.39^aB^
		SD	0.63	0.21
		Min	0.66	0.05
		Max	2.53	0.70
LH plasma	10	x¯	1.85^bA^	0.43^aB^
		SD	0.59	0.08
		Min	0.81	0.12
		Max	2.63	0.71
Friedman's test		*p*	<.001	0.789

*Note*: *p* = probability value of Friedman's test assessing possible differences between sample types. ^a,b^ Means of different lowercase superscript letters within a column differ significantly (Friedman's test; *p* < .05). ^A,B^ Means of different uppercase superscript letters within a row differ significantly between measurement techniques (Wilcoxon's test; *p* < .05).

Abbreviations: max, maximum; min, minimum; SD, standard deviation of the mean; x¯, mean.

**TABLE 2:** Results of a simple linear regression analysis assessing the relationships between haptoglobin (Hp) concentrations measured in serum with a commercial ELISA test kit (x) versus those determined with plasma derived from lithium‐heparinized (LH) tubes and EDTA tubes, respectively, using either the ELISA or a colorimetric assay (CA) on a chemical autoanalyzer (y).

*y*	Serum (ELISA) (*x*)			
	*B*	SE	*r*_p_	*p*
LH (ELISA)	0.86	0.057	.908	<.001
EDTA (ELISA)	0.88	0.084	.840	<.001
Serum (CA)	1.42	0.129	.828	<.001
LH (CA)	3.53	0.313	.862	<.001
EDTA (CA)	3.29	0.318	.795	<.001

*Note*: *n* = 10 pooled samples of each sample type from 100 dairy cows within 0–8 days postpartum from 10 farms. Simple linear regression analysis: *y* = *B* * *x*.

Abbreviations: *B*, unstandardized regression coefficient; *p*, probability value of Pearson's correlation; *r*_p_, Pearson's correlation coefficient; SE, standard error of *B*.

**TABLE 3:** Results of a simple linear regression analysis assessing the relationships between haptoglobin (Hp) concentrations measured in serum with a colorimetric assay (CA) on a chemical autoanalyzer (x) and those determined with plasma derived from lithium‐heparinized (LH) tubes and EDTA tubes, respectively, using either the CA or a commercial ELISA test kit (y).

*y*	Serum (CA) (*x*)			
	*B*	SE	*r*_p_	*p*
Serum (ELISA)	0.66	0.060	.828	<.001
LH (ELISA)	0.59	0.031	.923	<.001
EDTA (ELISA)	0.60.	0.033	.911	<.001
LH (CA)	2.48	0.058	.979	<.001
EDTA (CA)	2.33	0.072	.959	<.001

*Note*: *n* = 10 pooled samples of each sample type from 100 dairy cows within 0–8 days postpartum from 10 farms. Simple linear regression analysis: *y* = *B* * *x*.

Abbreviations: *B*, unstandardized regression coefficient; *p*, probability value of Pearson's correlation; *r*_p_, Pearson's correlation coefficient; SE, standard error of *B*.

Correlations among all three sample types were strong for the CA (Supplementary Table S1), whereas Hp concentrations determined in EDTA plasma samples were only weakly correlated with those determined in serum and LH plasma, respectively, when using the ELISA (Supplementary Table S1).

**FIGURE 1:**
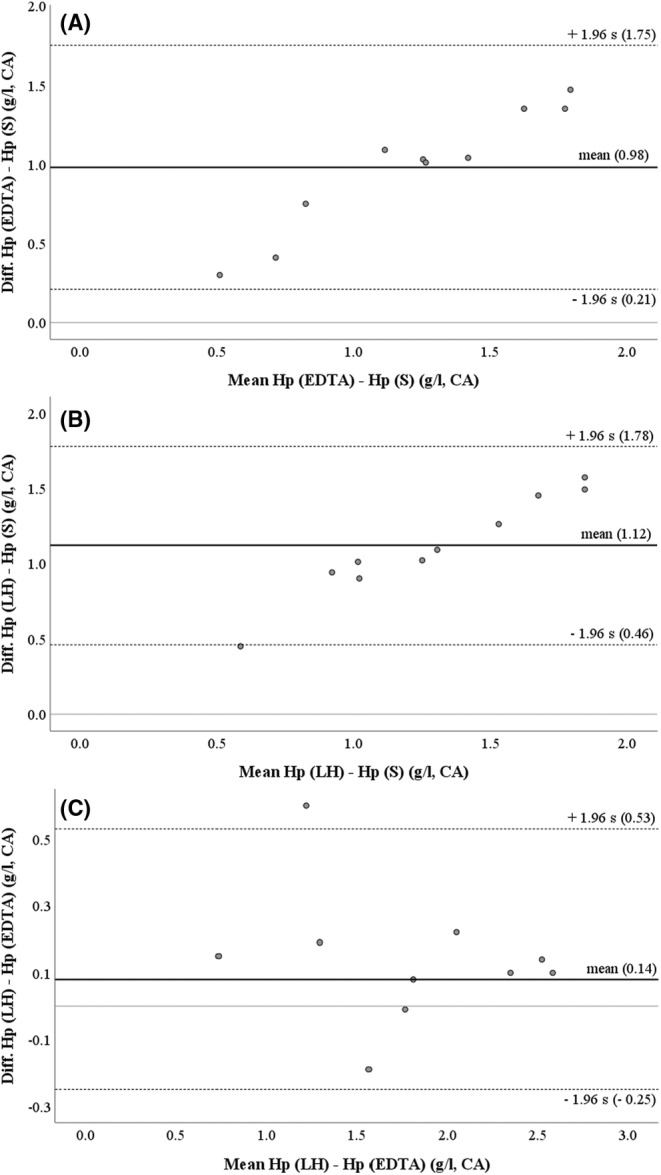
Bland–Altman plots showing the relationships between haptoglobin (Hp) concentrations determined by a chemical autoanalyzer (CA) in serum (S) and EDTA plasma (A), S and lithium‐heparinized (LH) plasma (B), and EDTA and LH plasma (C), respectively. ‐‐‐ = upper and lower limits of 95% confidence interval, — = mean of difference, *n* = 10 pooled aliquots of each sample type from 100 dairy cows within 0–8 days postpartum from 10 different farms.

The differences between measurement results of serum and both plasma variants using the CA increased with rising Hp concentrations in a linear pattern (Figure 1A,B). Between EDTA and LH plasma, with some inconsistency, a rather random deviation was observed (Figure 1C).

#### 3.1.2 Comparison of the CA and ELISA techniques

Mean Hp concentration determined in pooled serum, EDTA plasma, and LH plasma samples of transition cows from 10 farms differed significantly between the two measurement methods. Compared with the CA, the ELISA revealed on average 36.5%, 77.3%, and 76.8% lower values in serum (CA: 0.74 ± 0.25 g/L, ELISA: 0.47 ± 0.24 g/L; *p* < .05), EDTA plasma (CA: 1.72 ± 0.63 g/L, ELISA: 0.39 ± 0.21 g/L; *p* < .05), and LH plasma (CA: 1.85 ± 0.59 g/L, ELISA: 0.43 ± 0.08 g/L; *p* < .05), respectively (Table 1).

**FIGURE 2:**
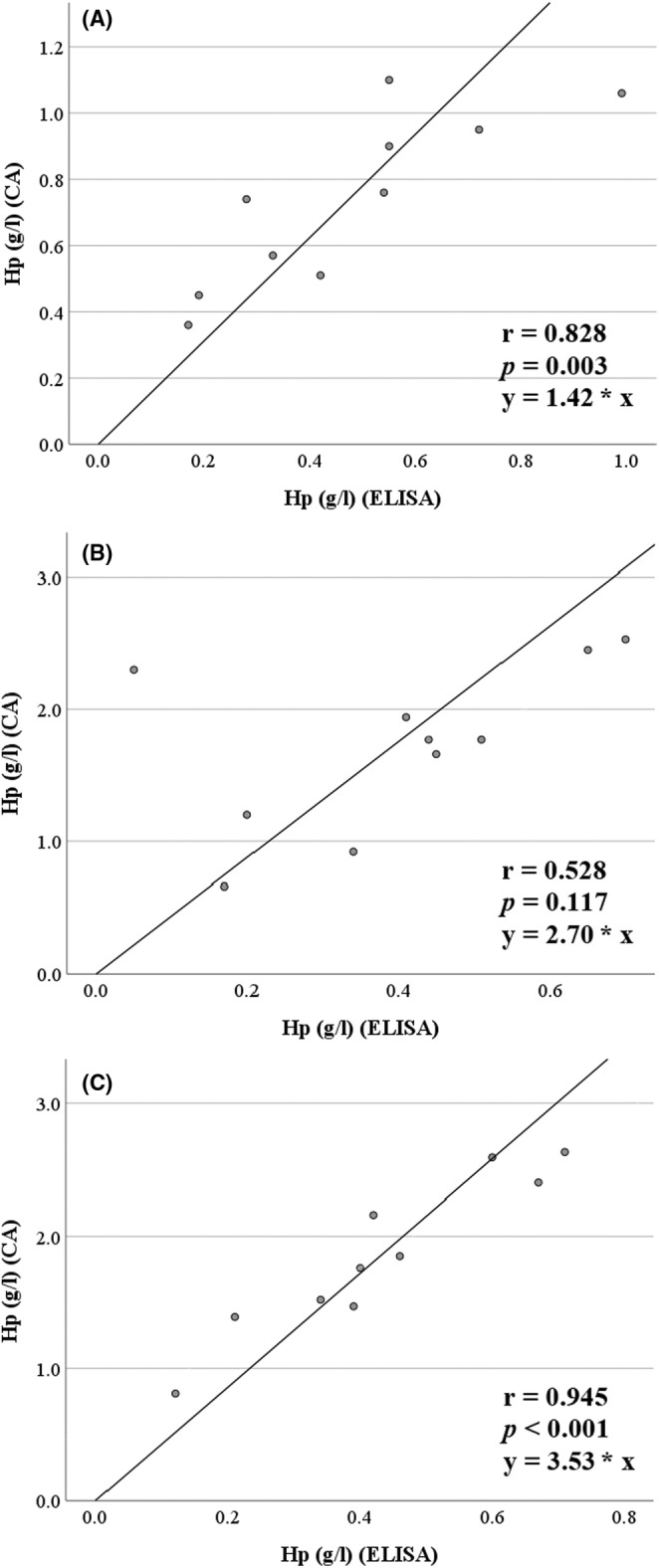
Scatterplots illustrating the association between haptoglobin (Hp) concentrations in pooled serum (A; *n* = 10), EDTA plasma (B; *n* = 10), and lithium‐heparinized (LH) plasma (C; *n* = 10) samples of dairy cows within 0–8 days postpartum determined using an ELISA test kit and a chemical autoanalyzer (CA), respectively.

Besides the observed numerical differences, measurement results of both methods were closely correlated when using serum (*r* = .828, *p* = .003) and LH plasma (*r* = .945, *p* < .001), respectively (Supplementary Table S1; Figure 2A and C). With EDTA plasma, the correlation between the two measurement techniques was not significant (*r* = .528, *p* = .117) (Supplementary Table S1; Figure 2B).

**FIGURE 3:**
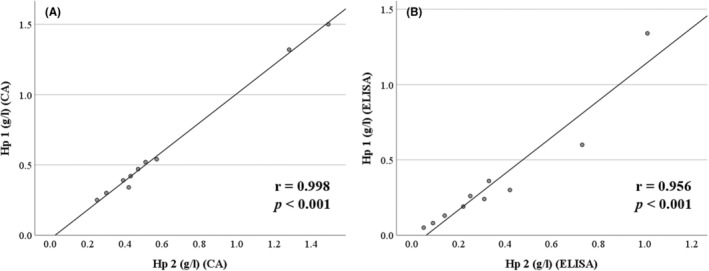
Scatterplots showing the association between serum duplicate analyses of haptoglobin (Hp) using either a colorimetric assay (CA) on a chemical autoanalyzer (A) or a commercial ELISA test kit (B) (*n* = 10 individual serum samples of dairy cows within 0–8 days postpartum from 10 farms). Intra‐assay CV: A: 1.75, B: 9.26 (calculated from *n* = 10 samples with the root mean square method).

**FIGURE 4:**
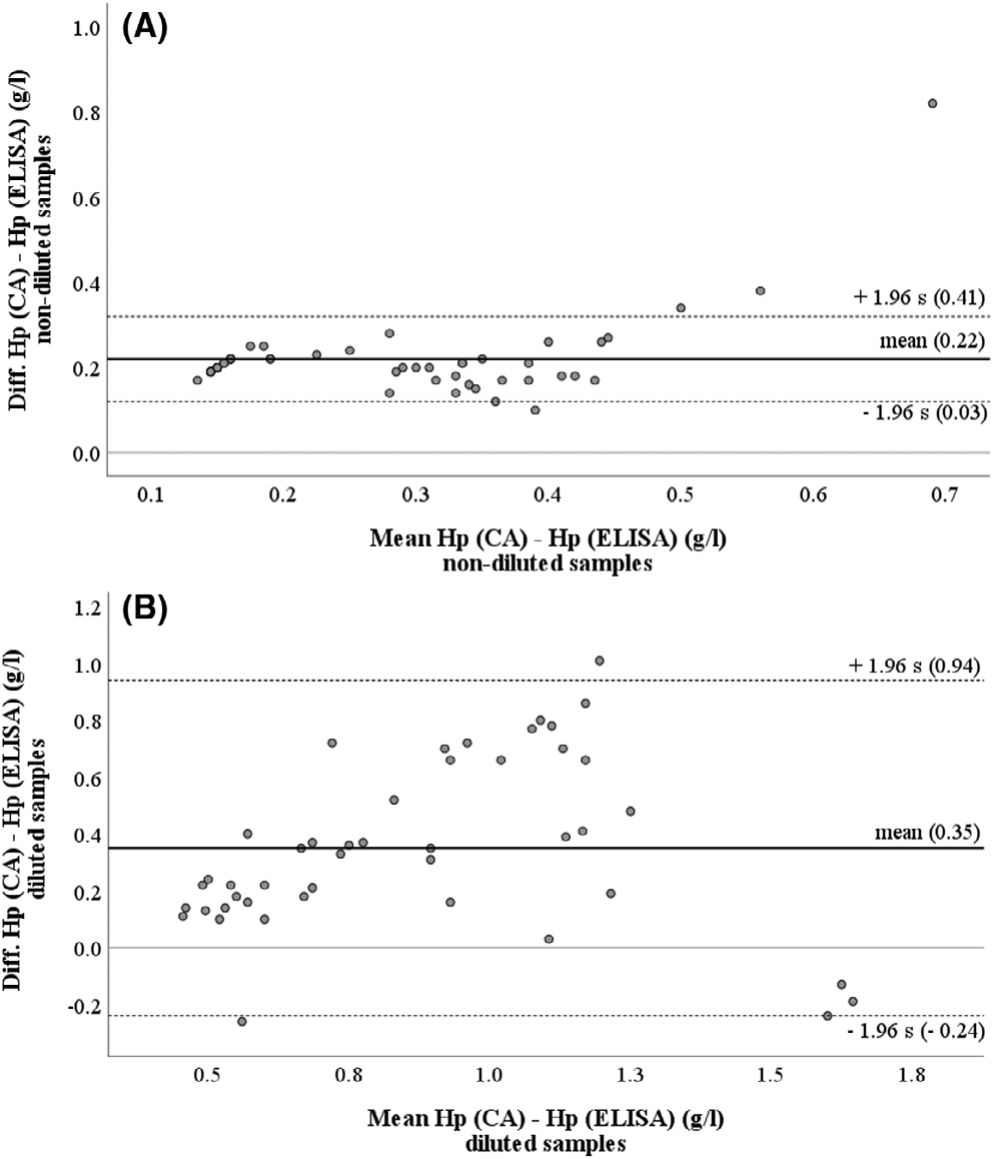
Bland–Altman plots illustrating the differences between the chemical autoanalyzer (CA) and the ELISA, including only those specimens measurable by ELISA without previous dilution (A) and including all specimens containing high amounts of haptoglobin (Hp) that needed to be diluted before measurement by the ELISA (B), respectively. ‐‐‐ = upper and lower limits of 95% confidence interval, — = mean of difference, *n* = 100 individual serum samples of dairy cows within 0–8 days postpartum from 10 different farms.

Serum duplicate measurements further revealed a higher precision of the CA (intra‐assay CV: 1.75%) compared with the ELISA (intra‐assay CV: 9.26%) (Figure 3A,B). Samples with Hp concentrations exceeding the measurement range of the ELISA (> 0.36 g/L) had to be further diluted. Comparing the results of nondiluted and further diluted serum samples of all cows, it becomes obvious that the process of dilution impaired the analytical precision of the ELISA: In nondiluted samples (Figure 4A), the mean of the difference between the two methods was 0.22 g/L, the 95% confidence interval ranged from 0.03 to 0.41 g/L, and did not include the zero line. In further diluted samples (Figure 4B), the mean difference was 0.35 g/L, the 95% confidence interval was much larger (−0.24 to 0.94 g/L), and it did include the zero line.

### 3.2 Experiment 2

It should be noted first that some important findings from experiment 1 were reproduced in experiment 2. These findings were that (1) Hp concentrations determined with the CA were on average 2.3 and 2.5 times higher in EDTA and LH plasma samples, respectively, compared with serum (Table 4; *p* < .001), (2) no differences between sample types were observed when using the ELISA test kit (Table 4; *p* = .867), and (3) Hp concentrations measured with the ELISA test kit were significantly lower compared with the CA (Table 4; *p* < .05).

**TABLE 4:** Haptoglobin measurements in serum, EDTA plasma, and lithium‐heparinized (LH) plasma from healthy (*n* = 5) and sick (metritis, *n* = 5) cows within 0–8 days postpartum using a colorimetric assay (CA) on a chemical autoanalyzer and a commercial ELISA test kit, respectively.

Assay	Sample type	Haptoglobin (g/L)			Mann–Whitney U test	Cut‐off applicable
		Healthy	Sick	Overall	*p*	(0.60 g/L Hp)^1^
CA	Serum	0.28 ± 0.07	1.41 ± 0.03	0.85 ± 0.19^aA^	.008	Yes
	EDTA plasma	1.37 ± 0.12	2.69 ± 0.16	2.03 ± 0.24^bA^	.008	No
	LH plasma	1.48 ± 0.13	2.86 ± 0.14	2.17 ± 0.25^bA^	.008	No
Friedman's test	*p*			<.001		
ELISA	Serum	0.18 ± 0.08	0.89 ± 0.09	0.53 ± 0.13^aB^	.008	Yes
	EDTA plasma	0.31 ± 0.19	0.89 ± 0.12	0.59 ± 0.14^aB^	.056	Yes
	LH plasma	0.22 ± 0.12	0.92 ± 0.10	0.57 ± 0.14^aB^	.016	Yes
Friedman's test	*p*			.867		

*Note*: Haptoglobin values are presented as mean ± standard error of the mean. ^a,b^ Means of different lowercase superscript letters within assay type differ significantly between sample types (Friedman's test). ^A,B^ Means of different uppercase superscript letters differ significantly between assay types (Wilcoxon's test; *p* < .05). ^1^ Cut‐off value (0.60 g/L Hp) based on available literature distinguishing early postpartum cows (within 0–8 days postpartum) with metritis from cows without metritis.^36^, ^37^

#### 3.2.1 Evaluating the ability of these assays to distinguish between sick and healthy cows

With the CA, Hp concentrations in all three sample types statistically differentiated sick from healthy cows (Table 4; *p* = .008). Serum samples analyzed with the CA matched the literature‐based clinical cut‐off value very well, with healthy animals below and sick animals above 0.60 g/L Hp. Contrary to this, the cut‐off value was widely surpassed by both types of plasma samples, regardless of the animal's health status. When using the ELISA, the cut‐off value was met with all three sample types. However, Hp concentrations in EDTA plasma samples were inconsistent (see standard deviation) and, hence, statistically not able to distinguish between sick and healthy cows (Table 4; *p* = .056). We further observed some inconsistency in Hp concentrations measured in LH plasma samples with the ELISA (based on the standard deviation), but differentiation between sick and healthy cows was still statistically significant (Table 4; *p* = .016).

### 3.3 Experiment 3

As observed in experiments 1 and 2, Hp concentrations measured with the CA differed significantly between serum and LH plasma. Here, the mean difference was greater with 4.8 times higher Hp values in LH plasma versus serum (Supplementary Table S2).

#### 3.3.1 Evaluation of possible influences of the anticoagulants on the colorimetric measurement

Despite some variability in the data, mixing of serum and LH plasma samples within plain, EDTA‐coated, and LH‐coated tubes prior to colorimetric analysis did not significantly affect Hp concentrations (Supplementary Table S2 and Figure S3; *p* = .074 for serum and *p* = .174 for LH plasma). In aliquots of distilled water previously mixed within the different types of tubes, the chemical autoanalyzer displayed Hp concentrations of 0.01 (serum and EDTA plasma) and 0.09 g/L (LH plasma), which appears rather insignificant (Supplementary Table S2 and Figure S3).

## 4 DISCUSSION

This study was designed to answer important methodologic questions regarding the laboratory analysis of bovine Hp in blood samples. Our primary hypotheses were that (1) Hp concentrations determined in serum and two different types of plasma (EDTA and LH) are the same and (2) Hp measurement results obtained from two commercially available assays, an ELISA test kit and a CA, as well as their diagnostic viability, are equal. To evaluate these hypotheses, two main experiments were designed, including all three sample types and the two measurement methods in different settings, allowing for both the replication of results and interpretation of the differentiated results. Hp analyses were conducted in accredited veterinary medical laboratories as part of their routine work.

To the authors' knowledge, this is the first study reporting diagnostically relevant differences in Hp concentration between serum and plasma when analyzed with a CA, which was not observed with the ELISA test kit. Although all three sample types were statistically able to differentiate sick from healthy cows when using the CA, Hp concentrations measured in plasma samples showed greater variability compared with serum samples and widely exceeded a clinical diagnostic cut‐off value, which is currently used in practice, regardless of the sample material. With the ELISA test kit, sick cows could be differentiated from healthy cows both statistically and from a clinical point of view (cut‐off based). However, although closely correlated, measurement results were significantly different between the two methods with lower values obtained from the ELISA test kit compared with the CA. Additionally, the ELISA was less precise than the CA due to dilutions. After having discovered clinically relevant differences in Hp concentrations between serum and plasma samples with the CA (and not with the ELISA), we added a small third experiment to the study, aiming to evaluate possible interfering effects of the anticoagulants EDTA and LH on the CA. Neither of the anticoagulants EDTA and LH was observed to directly influence the colorimetric measurement technique.

### 4.1 Comparison of serum, EDTA plasma, and LH plasma

The concentrations of Hp determined with the CA in both plasma variants were consistently higher and showed larger standard deviations and scattering compared with serum. The results from the ELISA test kit did not correspond to these findings, as there was no significant difference between the three sample types. Thus, we presume that the sample type somehow influences the colorimetric measurement method based on the peroxidase activity of Hp‐bound hemoglobin. Hussein et al.^26^ found higher values in plasma compared to serum analyzing the APP ceruloplasmin with a colorimetric assay, however, the reason for this phenomenon was likely a loss of detectable ceruloplasmin due to coagulation in serum tubes. This can be ruled out in this study, because if Hp had been captured within the coagulation clot, Hp concentrations determined with the ELISA test kit would have been lower in serum compared with plasma. Other authors have reported higher values of different chemical analytes in heparinized plasma compared to serum before,^38^, ^39^ but to our knowledge, this has not yet been assessed for bovine Hp. In their aforementioned study comparing a colorimetric method and an ELISA test kit for Hp analysis, Cooke and Arthington^25^ used heparinized plasma. The difference between the two methods was reported as 56% higher values (CA vs. ELISA). This percentage is located within the 36.5% difference between serum samples and the 77% difference between LH plasma samples observed in our study. Taking into consideration expectable data variability between the two studies, it might corroborate our finding that higher Hp concentrations are determined in LH plasma compared to serum when using a colorimetric measurement method. Double determinations showed that the colorimetric measurement is repeatable and precise, hence, internal errors of the method can be neglected. A possible explanation for the discrepancies between the sample materials might be related to the coagulation process in serum tubes, which is inhibited by EDTA and LH. Thus, remaining plasma components in EDTA‐ and LH‐coated tubes might influence peroxidase activity measured by the analyzer. A similar effect has been found in milk samples containing lactoperoxidase,^40^ however, it is not clear which substance would have similar effects in plasma. A possible interference of free hemoglobin that may have leaked from red blood cells into plasma during the time from sampling to centrifugation should be taken into consideration and cannot be fully ruled out in this study. However, according to the manufacturer of the CA, only grossly hemolysed samples containing >2.5 g/L hemoglobin should be excluded from analyses as test results may not be reliable, while mild hemolysis is declared as irrelevant. In this study, only samples that were macroscopically free from hemolysis^41^ were used for Hp analyses, thereby decreasing the risk of interference by hemoglobin.

Another conceivable reason for the differences between serum and plasma could have been an interference of the respective anticoagulant itself with the colorimetric measurement technique. Pure heparin was found to enhance peroxidase‐like activity of gold nanoclusters in another study,^42^ however, this interaction is very specific and it is not clear how LH would possibly interfere with the peroxidase activity detected by the colorimetric Hp assay used in this study. In experiment 3 of this study, we did not find any hints toward effects of the anticoagulants themselves previously mixed with serum and LH plasma samples, respectively, on the results of the colorimetric Hp assay. Using distilled water previously mixed with LH and EDTA as a control, we were further able to show that neither anticoagulant affected the measurement to a degree that would explain the differences between serum and plasma. It should be noted here that the sample size for experiment 3 was severely limited. Nevertheless, the close linear relationships between the values obtained using both plasma variants and serum reported in this study support the conclusion that the anticoagulant itself is unlikely to be the cause of the differences, since LH and EDTA would most likely react differently. Hence, the cause of the high Hp values found in plasma samples compared to serum when using the CA might be a certain plasma component expressing or enhancing peroxidase activity, but this remains speculative for the time being. Further research is needed to find an explanation for this phenomenon.

### 4.2 Comparison of the CA and ELISA techniques

The observed fact that the colorimetric Hp assay revealed significantly higher values compared to the ELISA coincides with the findings of another study.^25^ There, an APR was triggered in nine mixed breed steers by vaccination against *Mannheimia haemolytica*. The colorimetric method based on peroxidase activity revealed on average 56% higher Hp concentrations when compared to an ELISA, but the results from both methods were positively and closely correlated (*r* = .97, *p* < .01), as in this study. Methodological differences between the two methods (i.e., true Hp concentrations of standards and controls used, number and extent of dilutions required, number, range, and concentration of calibrator solutions used to establish the calibration curve) are most likely the reason for the numerical differences in detection range. It should be mentioned here that the source of Hp used in the respective calibrator solutions was different among the two assays (CA: equine serum, ELISA: bovine serum). However, due to a high interspecies homology of the Hp molecule, especially regarding the hemoglobin‐binding site, there should be no difference in hemoglobin binding among species.^42^, ^43^ Because the Hp value determined spectrophotometrically with the CA is based on the peroxidase activity of Hp‐bound hemoglobin, the fact that equine Hp was used in calibrator solutions is unlikely to have caused the observed numerical differences in detection range, but it cannot be fully ruled out. Aside from differences in detection range, ELISA measurements in this study were less precise when additional dilutions were performed due to high concentration of Hp in a specimen. These findings underline the importance of accurate and corrected dilutions to obtain reliable results. Considering the capability of both assays to differentiate between sick and healthy cows shown in this and other studies,^8^, ^9^, ^44^ it can be stated that both methods are suitable for the measurement of Hp concentrations and, hence, for the diagnosis of inflammatory reactions in cattle. However, latest research assessing Hp as clinical diagnostic marker in postpartum dairy cow pathology reveals cut‐off values for sick versus healthy cows that are consistently >0.36 g/L (i.e., 0.60 g/L as presented in [36, 37]), thereby exceeding the measurement range of the ELISA. Acknowledging the higher measurement precision of the CA in this range of Hp concentrations, especially when performed with serum samples, the latter should be the preferred measurement technique for studies in the peripartal setting. In general, it could be helpful for veterinary practitioners if method‐specific (and, if necessary, sample type‐specific) reference values (or at least a specific declaration of the required sample type) for the analysis of bovine Hp were provided by laboratories.

## 5 CONCLUSIONS

In this study, bovine Hp concentrations were repeatedly and relevantly higher in both EDTA and LH plasma samples and showed higher variability (especially EDTA) compared with serum when analyzed with a commercially available CA. This was not observed with an ELISA test kit. The reason for this phenomenon remains unclear and warrants further research. An influence of the anticoagulants EDTA and LH themselves was found unlikely in this study. Acknowledging the current lack of clarity and considering the results presented here, it is recommended to perform colorimetric Hp assays based on peroxidase activity with serum samples instead of plasma or to determine specific reference intervals and cut‐off values when using EDTA or LH plasma. The ELISA can be performed with all three sample types tested, but due to slight inconsistencies observed with EDTA plasma, serum and LH plasma are preferred. For the analysis of bovine Hp from serum samples, aiming to assess the degree of systemic inflammation, the CA and the ELISA tested in this study provided reliable results despite different measurement ranges. The dilution of specimens containing high amounts of Hp is inevitable using the ELISA technique and can impair measurement precision. Specimens must be prepared with a high level of accuracy to provide reliable results. Because common clinical cut‐off values used to differentiate sick from healthy dairy cows in the periparturient period exceed the measurement range of the ELISA, the CA (performed using serum samples) is the preferred technique for this purpose.

## CONFLICT OF INTEREST STATEMENT

There are no conflicts of interest that could inappropriately bias the content of this article.

## Supporting information


**Data S1.** Supporting Information.
