# Supplementary material for: Discrepancies of bovine haptoglobin concentrations between serum and plasma using two different anticoagulants and a colorimetric assay based on peroxidase activity
Source: Vet Clin Pathol. 2024 Oct 21;53(4):409–19. doi: 10.1111/vcp.13386 (PMC11735647; doi:10.1111/vcp.13386)
Supplement: Supplementary file 1 — Data S1. Supporting Information. [file VCP-53-409-s001.docx]

# SUPPLEMENTARY TABLES

**Supplementary Table S1.** Correlation matrix of haptoglobin concentrations determined in pooled serum (S, n = 10), EDTA plasma (n = 10), and lithium heparinized (LH, n = 10) plasma samples of n = 100 dairy cows within 0 – 8 days postpartum from 10 different farms using a colorimetric assay (CA) on a chemical autoanalyzer, and a commercial ELISA test kit, respectively.

|  |  | **S (CA)** | **EDTA (CA)** | **LH (CA)** | **S (ELISA)** | **EDTA (ELISA)** | **LH (ELISA)** |
| --- | --- | --- | --- | --- | --- | --- | --- |
| **S (CA)** | **r_p_** | - | 0.959 | 0.979 | 0.828 | 0.548 | 0.923 |
|  | ***p*** | - | < 0.001 | < 0.001 | 0.003 | 0.101 | < 0.001 |
| **EDTA (CA)** | **r_p_** | 0.959 | - | 0.949 | 0.795 | 0.528 | 0.933 |
|  | ***p*** | < 0.001 | - | < 0.001 | 0.006 | 0.117 | < 0.001 |
| **LH (CA)** | **r_p_** | 0.979 | 0.949 | - | 0.862 | 0.509 | 0.945 |
|  | ***p*** | < 0.001 | < 0.001 | - | 0.001 | 0.133 | < 0.001 |
| **S (ELISA)** | **r_p_** | 0.828 | 0.795 | 0.862 | - | 0.446 | 0.908 |
|  | ***p*** | 0.003 | 0.006 | 0.001 | - | 0.197 | < 0.001 |
| **EDTA (ELISA)** | **r_p_** | 0.548 | 0.528 | 0.509 | 0.446 | - | 0.463 |
|  | ***p*** | 0.101 | 0.117 | 0.133 | 0.197 | - | 0.177 |
| **LH (ELISA)** | **r_p_** | 0.923 | 0.933 | 0.945 | 0.908 | 0.463 | - |
|  | ***p*** | < 0.001 | < 0.001 | < 0.001 | < 0.001 | 0.177 | - |

r_p_ = Pearson’s correlation coefficient, *p* = Probability value of Pearson’s correlation

**Supplementary Table S2.** Colorimetric haptoglobin measurement results in serum and lithium heparinized (LH) plasma samples from 4 cows in different lactation stages, and distilled water, respectively, each aliquot (1 ml) previously mixed within three different types of tubes (plain tube, LH-coated tube, and EDTA-coated tube).

|  |  | **Haptoglobin (g/l) in Sample Type** | | |
| --- | --- | --- | --- | --- |
| **Tube type** | **Sample ID** | **Serum** | **LH** | **Distilled Water** |
| **Plain tube** | **Cow 1** | 0.33 | 1.20 |  |
|  | **Cow 2** | 0.13 | 0.78 |  |
|  | **Cow 3** | 0.16 | 0.43 |  |
|  | **Cow 4** | 0.14 | 1.22 |  |
|  | **Mean** | 0.19 ± 0.05^aA^ | 0.91 ± 0.19^aB^ |  |
|  | **Control** |  |  | 0.01 |
| **LH tube** | **Cow 1** | 0.34 | 0.74 |  |
|  | **Cow 2** | 0.14 | 0.92 |  |
|  | **Cow 3** | 0.15 | 0.67 |  |
|  | **Cow 4** | 0.19 | 1.12 |  |
|  | **Mean** | 0.21 ± 0.05^aA^ | 0.86 ± 0.10^aB^ |  |
|  | **Control** |  |  | 0.09 |
| **EDTA tube** | **Cow 1** | 0.39 | 0.54 |  |
|  | **Cow 2** | 0.16 | 0.58 |  |
|  | **Cow 3** | 0.16 | 0.48 |  |
|  | **Cow 4** | 0.20 | 0.91 |  |
|  | **Mean** | 0.23 ± 0.06^aA^ | 0.63 ± 0.10^aB^ |  |
|  | **Control** |  |  | 0.01 |
| **Friedman’s test** | ***p*** | 0.074 | 0.174 |  |

Haptoglobin values are presented as raw measurement results, and as mean ± standard error of the mean, respectively.

^a^ previous mixing within different tubes did not affect haptoglobin measurements (Friedman’s test)

^A, B^ means of different uppercase superscript letters differ significantly between serum and LH plasma (Wilcoxon’s test; *p* < 0.05)

# SUPPLEMENTARY FIGURE


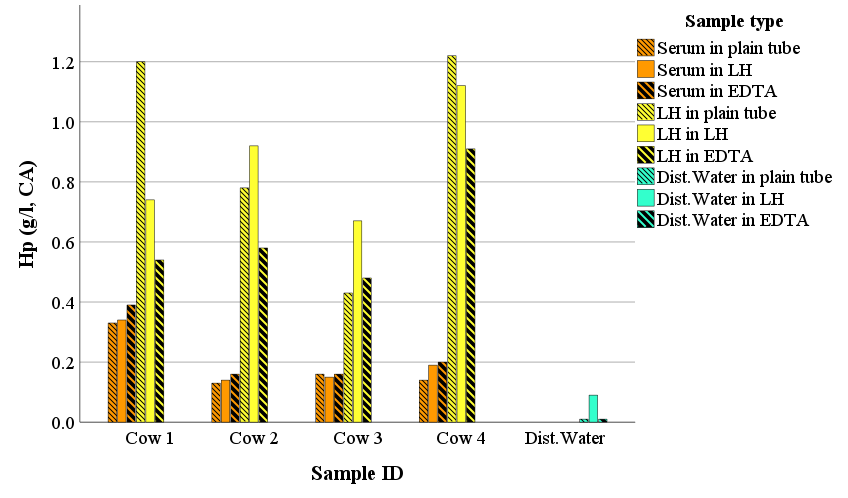


**Supplementary Figure S3.** Haptoglobin (Hp) concentrations in cows from different lactation stages (1 = early postpartum, 2 = mid lactation, 3 = late lactation, 4 = dry period), and distilled water (Dist. Water), respectively, determined using a colorimetric assay (CA) on a chemical autoanalyzer in serum and lithium heparinized (LH) plasma, each previously mixed within three different types of tubes (plain tube, LH-coated tube, and EDTA-coated tube).
